# Casual alcohol consumption is associated with less subclinical cardiovascular organ damage in Koreans: a cross-sectional study

**DOI:** 10.1186/s12889-018-6000-x

**Published:** 2018-09-04

**Authors:** Jeonggeun Moon, In Cheol Hwang, Kyoung Kon Kim, Woong Chol Kang, Ji-Young Cha, Young-Ah Moon

**Affiliations:** 1 Cardiology Division, Department of Internal Medicine, Gil Medical Center, Gachon University College of Medicine, Incheon, Republic of Korea; 2 Department of Family Medicine, Gil Medical Center, Gachon University College of Medicine, Incheon, Republic of Korea; 3 Department of Biochemistry, Lee Gil Ya Cancer and Diabetes Institute, Gachon University College of Medicine, Incheon, Republic of Korea; 4 Gachon Medical Research Institute, Gil Medical Center, Gachon University College of Medicine, Incheon, Republic of Korea; 5 Department of Molecular Medicine, Inha University School of Medicine, 100 Inha-ro, Nam-gu, Incheon, 22212 Republic of Korea

**Keywords:** Alcohol, Pulse wave velocity, Coronary artery disease, Atherosclerosis, Arteriosclerosis

## Abstract

**Background:**

Epidemiologic studies have presented protective effects of alcohol against cardiovascular (CV) events. However, such studies were performed mainly on Westerners. We investigated the effects of alcohol on the subclinical CV morbidity in healthy Koreans.

**Methods:**

The coronary artery calcium (CAC) score, ankle-brachial pulse wave velocity (abPWV), and carotid intima-media thickness (cIMT) of 1004 subjects (age, years±standard deviation [SD] 53 ± 10; 72% were men) with no CV disease history were assessed. The subjects were divided into three groups based on their drinking patterns: Group 0 (abstainers), Group 1 (casual drinkers), and Group 2 (problematic drinkers; &gt; 14 standard drinking/week for men, &gt; 7 standard drinking/week for women). As drinking patterns can be influenced by age/sex, a regression analysis was performed in another four groups (men/women, age &lt; 65/≥65 years).

**Results:**

Group 1 exhibited lower CAC (score ± SD, 44 ± 155 vs. 13 ± 48 vs. 50 ± 159) and abPWV (cm/s ± SD, 1448 ± 284 vs. 1340 ± 190 vs. 1447 ± 245) scores and thinner cIMT (mm ± SD, 0.64 ± 0.14 vs. 0.59 ± 0.11 vs. 0.63 ± 0.13) than Groups 0 and 2 (*p* &lt; 0.05 for all). Problematic drinking (odds ratio [OR]: 2.269; 95% confidence interval [CI]: 1.454–3.541) was associated with a high prevalence of CAC deposits among men aged &lt; 65 years and casual drinking with a lower prevalence of CAC deposits (OR: 0.057; 95% CI: 0.023–0.140) among men aged ≥65 years. Conversely, problematic drinking in older women [OR: 0.117; 95% CI: 0.014–0.943) and casual drinking in younger women (OR: 0.349; 95% CI: 0.153–0.792) were associated with a lower prevalence of CAC deposits. Casual drinking was associated with a lower abPWV and thinner cIMT in the diabetes mellitus/hypertension-adjusted regression analysis.

**Conclusions:**

Compared with abstinence or problematic drinking, casual drinking was associated with less severe CV organ damage in the subclinical stages in Koreans.

**Electronic supplementary material:**

The online version of this article (10.1186/s12889-018-6000-x) contains supplementary material, which is available to authorized users.

## Background

The effects of alcohol on cardiovascular (CV) diseases are still controversial, although various epidemiologic observational studies have demonstrated cardioprotective effects of alcohol, which has been known as the “French paradox” [1–5]. The chronic effects of habitual light-to-moderate drinking (i.e., “casual drinking” in this study) need to be elucidated in a long-term longitudinal study, ideally a randomized controlled trial. However, such studies are difficult to perform, and the end-point of clinical CV diseases occurs at late stages in life. With the recent advances in diagnostic tools, early manifestations of CV morbidities can be noninvasively assessed even in the subclinical stage. Among these, assessment of coronary artery calcium (CAC) deposits using computed tomography (CT) can screen significant coronary artery plaques with a minimal radiation dose; the pulse wave velocity (PWV) serves as a marker for arterial stiffening; and the carotid intima-media thickness (cIMT) is used not only as an early indicator of subclinical organ damage but also a predictor of future atherosclerosis. These parameters are usually obtained from apparently healthy subjects during a health check-up process in Republic of Korea.

Whether drinking alcohol is beneficial to CV morbidities and whether these beneficial effects are from alcohol itself or from other substances contained in alcoholic beverages, such as antioxidants in wine and beer, remain to be elucidated [5]. To answer these, data from different countries with diverse ethnic groups, drinking habits, and unique liquors would be informative. The Korean population has a distinct drinking culture with liquors different from those in Western populations. The most frequently consumed alcoholic beverage in Korea is soju, a type of distilled alcohol, which contains almost zero antioxidants, such as polyphenol that is abundant in wine or beer [5–8]. Most previous studies on the effects of alcohol on CV diseases were performed in Western countries [9, 10]; their findings may not be applicable to non-Westerners. In this study, we performed cross-sectional analyses to investigate the effects of habitual alcohol drinking on the subclinical CV morbidity in healthy Korean subjects with no apparent CV diseases.

## Methods

### Study sample

The study protocol was approved by the institutional review board (IRB) of Gachon University Gil Hospital (GCIRB2018–098), and the study complied with the Declaration of Helsinki principles (6th revision). We used medical data and self-reported questionnaires of 1004 Korean subjects, which were obtained upon a health check-up between 2016 and 2017 in the attending hospital. The self-reported questionnaire contained quantitative drinking habits (standard drinking/week that had been maintained for &gt; 5 years before the visit), smoking history, and comorbidities, including diabetes mellitus (DM) and hypertension. Blood tests including fasting sugar, HbA1c, blood urea nitrogen/creatinine, liver panel, uric acid, and lipid panel were performed in all participants after a 12-h fast. We excluded patients with morbidities other than DM and hypertension, such as overt CV diseases and cancers. Problematic drinking was defined as &gt; 14 standard drinking/week for men and &gt; 7 standard drinking/week for women; the cut-off value in women has been defined as lower than that in men in other investigations because 1) women tend to weigh less than do men; 2) women’s bodies contain less water and more fats that retain alcohol. As water dilutes alcohol, it remains at higher concentrations for longer periods of time in a woman’s body, exposing her organs to more alcohol; 3) women have lower levels of alcohol dehydrogenase and aldehyde dehydrogenase; and 4) changes in hormone levels during the menstrual cycle may also affect alcohol metabolism in a woman’s body [3, 4]. Alcohol drinking that is less than that in the problematic range was designated as casual drinking (i.e., 1–14 standard drinking/week for men and 1–7 standard drinking/week for women) [9, 10]. Ex-drinkers were all excluded as biases were a concern, such as reverse causality unmeasured effect modification or residual confounding; and life-time abstainers were regarded as the “abstainers.” The cohort was divided into three groups: Group 0: abstainers, Group 1: casual drinkers, and Group 2: problematic drinkers. Drinking patterns are influenced by age and sex; therefore, we designated another four groups based on demographic data (men/women and age of &lt; 65/≥65 years) to assess the independent effects of alcohol on subclinical CV organ damage.

### CAC score, ankle-brachial PWV (abPWV), cIMT, and abdominal ultrasound findings

The CAC score was assessed in all subjects with electrocardiogram (ECG)-gated CT examinations in one session using a 64-channel multi-detector CT scanner (SOMATOM Definition or Sensation 64, Siemens Medical Solutions, Forchheim, Germany). The scanning protocol for calcium scoring using ECG-gated CT was as follows: 1) prospective ECG gating was used; 2) the peak voltage was 120 kVp; 3) the tube current-exposure time product was 40 mAs; 4) the rotation time was 0.36 s; 5) the collimation was 64 × 0.6 mm; 6) the pitch was 0.33; 7) the scan range was from the carina to the cardiac apex; 8) the field of view was 250 mm^2^; 9) the raw data were reconstructed at the early to the mid-diastolic phase; 10) the reconstruction algorithm was medium-sharp kernel (B35F); and 11) the slice thickness/increment was 3.0/1.5 mm. The image datasets acquired from the calcium scoring using CT were transferred to a dedicated workstation (Leonardo, Siemens Medical Solutions, Forchheim, Germany) to determine the CAC score. The presence of CAC deposits was defined as a CAC score &gt; 0 using the modified Agatston method.

The abPWV of 569 subjects was measured at their discretion, which was automatically determined using the oscillometric method with a commercially available volume plethysmography equipment (Colin VP-1000, Colin Medical Instrument Co., Komaki, Japan). To measure blood pressure (BP), cuffs were wrapped around both upper arms and ankles of the subjects in the supine position after a 5-min rest in a quiet room. Pulse waves from the brachial and tibial arteries were simultaneously obtained and recorded. The abPWV was calculated as the transmission distance (distance between the cuff position of the arm and the ankle) divided by the transmission time (interval between the initial increases in the brachial and tibial wave forms). We used the abPWV (mean of the right-side and left-side values) as a marker of both central and peripheral arterial stiffness.

The cIMT was measured in 562 subjects using ultrasound after obtaining their consent. The subjects rested in the supine position for 15 min, and their cIMT was measured using an 8-MHz linear array transducer (Sequoia 512; Acuson, Mountain View, CA, USA) and automatic IMT measurement software (M’Ath-Std; Metris, Argenteuil, France) on a computer. Images from the region 2 cm proximal to the carotid bifurcation were obtained at least 1 cm axially, and the distance between the lumen-intima interface and the media-adventitia interface was determined using an automated edge detection algorithm. The mean values of the right and left cIMTs were used for the analysis.

Abdominal ultrasound using a 3.5 MHz transducer was performed in all participants after a 12-h fast to diagnose fatty liver. The studies were conducted by two experienced radiologists who were blinded to the aims of the study and laboratory values; images were captured with the participants in the supine position with their right arm raised above their head. Known ultrasound criteria such as hepatorenal echo contrast, liver brightness, deep attenuation, and vascular blurring were assessed. Those presenting hepatorenal contrast and liver brightness were diagnosed as having fatty liver [11].

### Statistical analysis

Continuous data were expressed as means±standard deviations (SD) after a normality test. The baseline characteristics were compared using the one-way ANOVA test, and post hoc analyses were performed using the Bonferroni procedure. Categorical variables were compared using the chi-square test and Fisher’s exact test. Binary logistic regression (odds ratios [ORs] and 95% confidence intervals [CIs] were calculated), and linear regression analyses (beta-value was provided) were performed to assess the independent association of casual or problematic drinking compared to abstinence. The Statistical Package for the Social Sciences v18.0 (SPSS Inc., Chicago, IL, USA) was used, and *p-*values of &lt; 0.05 were considered significant.

## Results

The baseline characteristics of the cohort and the intergroup comparison of results are presented in Table 1. The subjects were divided into three groups based on their alcohol consumption patterns: Group 0, abstainers; Group 1, casual drinkers; and Group 2, problematic drinkers. The mean age of the subjects in Groups 1 and 2 was 50 ± 9 and 51 ± 9 years, respectively; the subjects in Groups 1 and 2 were younger than those in Group 0 (age, years±SD, 55 ± 11). The proportion of women (7%) was smaller and the proportion of smokers (73%) was significantly higher in Group 2 than in the other groups. Overall, approximately 9% of the subjects had DM, and 25% had hypertension. The systolic and diastolic BPs were lower in Group 1 than in Group 2. The total cholesterol and low-density lipoprotein (LDL)-cholesterol levels were not significantly different among the groups; conversely, the high-density lipoprotein (HDL)-cholesterol level was lower in Group 0 compared to Groups 1 and 2. The CAC score and abPWV were significantly lower and the carotid intima-media was thinner in Group 1 than in Groups 0 and 2. The carotid intima-media tended to be thinner in Group 1 than in Group 0. Fatty liver was detected using ultrasound; the prevalence was lower in Group 1 than in the other groups. However, the parameters associated with fatty liver, such as the aspartate transaminase, alanine transaminase, gamma-glutamyltransferase, triglyceride, uric acid, and fasting glucose levels, were significantly higher in Group 2 than in the other groups; they were not significantly different between Groups 0 and 1.

**Table 1:** Baseline characteristics and intergroup comparison

Variables	All (*n* = 1004)	Group 0 (*n* = 487)	Group 1 (*n* = 178)	Group 2 (*n* = 339)	*p*-value			
					all	Group 0 vs. Group 1	Group 0 vs. Group 2	Group 1 vs. Group 2
Clinical parameters								
Age, years	53 ± 10	55 ± 11	50 ± 9	51 ± 9	&lt; 0.001	&lt; 0.001	&lt; 0.001	0.539
Male sex, n (%)	721 (72)	330 (68)	77 (43)	314 (93)	&lt; 0.001	&lt; 0.001	&lt; 0.001	&lt; 0.001
Diabetes mellitus, n (%)	92 (9)	51 (10)	9 (5)	32 (9)	0.106	0.033	0.724	0.089
Hypertension, n (%)	254 (25)	124 (32)	30 (17)	100 (29)	0.008	0.022	0.204	0.002
Smoking, n (%)	487 (49)	181 (37)	59 (33)	247 (73)	&lt; 0.001	0.464	&lt; 0.001	&lt; 0.001
Body mass index (kg/m^2^)	25 ± 3	25 ± 3	24 ± 3	25 ± 3	&lt; 0.001	0.031	0.082	&lt; 0.001
Systolic BP (mmHg)	123 ± 14	123 ± 14	120 ± 16	125 ± 14	&lt; 0.001	0.034	0.042	&lt; 0.001
Diastolic BP (mmHg)	76 ± 11	75 ± 10	74 ± 11	80 ± 10	&lt; 0.001	0.131	&lt; 0.001	&lt; 0.001
Laboratory parameters								
Fasting glucose (mg/dL)	94 ± 23	93 ± 22	91 ± 22	97 ± 24	0.008	0.580	0.037	0.013
HbA1c (%)	5.7 ± 0.8	5.8 ± 0.9	5.6 ± 0.8	5.7 ± 0.7	0.191	0.164	0.847	0.369
Blood urea nitrogen (mg/dL)	14 ± 4	15 ± 4	14 ± 4	14 ± 4	0.207	0.229	0.462	0.794
Creatinine (mg/dL)	0.8 ± 0.3	0.8 ± 0.2	0.7 ± 0.2	0.9 ± 0.4	&lt; 0.001	0.027	0.004	&lt; 0.001
Aspartate transaminase (U/L)	29 ± 21	28 ± 14	26 ± 11	32 ± 32	0.001	0.528	0.004	0.002
Alanine transaminase (U/L)	33 ± 31	32 ± 26	28 ± 20	36 ± 39	0.011	0.293	0.125	0.010
Alkaline phosphatase (U/L)	62 ± 17	64 ± 17	61 ± 15	60 ± 17	0.003	0.073	0.004	0.923
Gamma-glutamyl transferase (U/L)	53 ± 76	38 ± 39	38 ± 62	83 ± 108	&lt; 0.001	0.993	&lt; 0.001	&lt; 0.001
Uric acid (mg/dL)	5.8 ± 1.6	5.6 ± 1.4	5.4 ± 1.4	6.3 ± 1.7	&lt; 0.001	0.250	&lt; 0.001	&lt; 0.001
Total cholesterol (mg/dL)	196 ± 38	193 ± 37	198 ± 41	197 ± 36	0.232	0.327	0.362	0.949
Triglyceride (mg/dL)	138 ± 107	125 ± 78	125 ± 126	163 ± 125	&lt; 0.001	0.998	&lt; 0.001	&lt; 0.001
High-density lipoprotein (mg/dL)	50 ± 12	49 ± 11	52 ± 13	51 ± 13	0.005	0.005	0.131	0.309
Low-density lipoprotein (mg/dL)	121 ± 36	121 ± 36	123 ± 39	119 ± 34	0.472	0.786	0.730	0.452
High-sensitivity CRP (mg/L)	0.15 ± 0.36	0.15 ± 0.34	0.14 ± 0.28	0.17 ± 0.42	0.531	0.959	0.617	0.597
Organ damage assessment								
Coronary calcium score	40 ± 144	44 ± 155	13 ± 48	50 ± 159	0.016	0.039	0.890	0.015
Ankle-brachial PWV (cm/s)	1429 ± 259	1448 ± 284	1340 ± 190	1447 ± 245	0.001	0.001	1.000	0.002
Carotid intima-media thickness (mm)	0.63 ± 0.13	0.64 ± 0.14	0.59 ± 0.11	0.63 ± 0.13	0.030	0.022	0.668	0.125
Fatty liver on ultrasound, n (%)	502 (50)	245 (50)	73 (41)	184 (54)	0.018	0.042	0.255	0.005

**Fig. 1:**
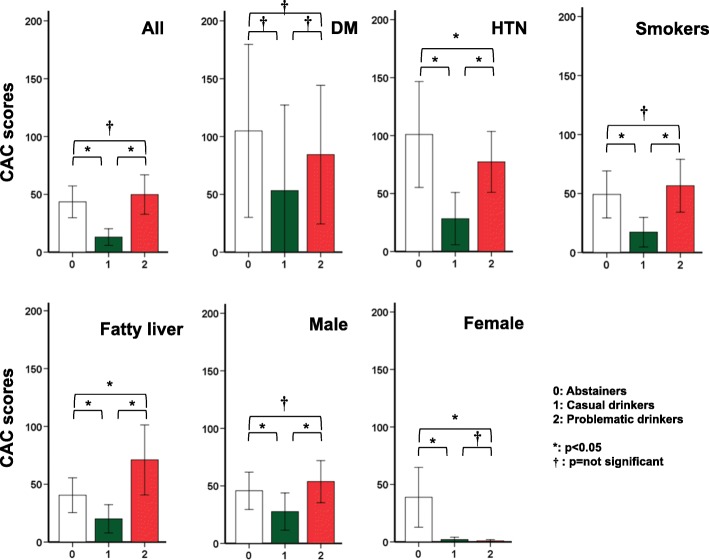
Intergroup comparison of the coronary artery calcium (CAC) score. CAC scores of groups 0 (abstainers), 1 (casual drinkers), and 2 (problematic drinkers) were compared using one-way ANOVA (*p* &lt; 0.05 for all) and post hoc analysis. The casual drinkers showed significantly lower CAC scores, and this phenomenon was also observed in the subjects with known risk factors of cardiovascular disease, such as diabetes mellitus, hypertension, smoking, and fatty liver

Figure 1 shows the comparison of CAC scores among Groups 0, 1, and 2, considering the known risk factors for atherosclerosis, such as DM, hypertension, smoking, fatty liver, and sex. The CAC score of Group 1 was lower than those of Groups 0 and 2 under all the risk factors, which corresponded to the “V-shape” (similar to the so-called “J-shape”), except in the female group.

As drinking pattern can be influenced by age and sex, we divided our cohort into four groups based on age and sex of the participants, and binary logistic regression analyses were performed to assess the predictive power of casual or problematic drinking for the presence of any CAC deposit. Known risk factors such as DM and hypertension were also considered (Table 2). In men aged &lt; 65 years, problematic drinking was associated with a higher prevalence of CAC deposits. In men aged ≥65 years, casual drinking was related to a lower prevalence of CAC deposits than no drinking. Conversely, the influence of problematic drinking on CAC deposits was not noticeable in women aged ≥65 years, presumably because the number of problematic drinkers in women was too small. The CAC deposits were rather less prevalent in problematic female drinkers aged &lt; 65 years. Among the women aged ≥65 years, casual drinking was rather correlated with a lower prevalence of CAC deposits even after adjusting for DM and hypertension.

**Table 2:** Binary logistic regression model for the presence of coronary calcium deposits on computed tomography to assess the independent effects of alcohol consumption

Variables	Unadjusted	*p*-value	Adjusted for DM and hypertension	*p*-value
	OR (95% confidence interval)		OR (95% confidence interval)	
Men, &lt; 65 years (*n* = 651)				
Abstainer	1.000		1.000	
Casual drinker	0.678 (0.437–1.050)	0.082	0.752 (0.478–1.182)	0.216
Problematic drinker	2.530 (1.655–3.868)	&lt; 0.001	2.269 (1.454–3.541)	&lt; 0.001
Men, ≥65 years (*n* = 70)				
Abstainer	1.000		1.000	
Casual drinker	0.116 (0.057–0.236)	&lt; 0.001	0.057 (0.023–0.140)	&lt; 0.001
Problematic drinker	3.462 (1.186–9.318)	0.014	2.497 (0.851–7.330)	0.096
Women, &lt; 65 years (*n* = 240)				
Abstainer	1.000		1.000	
Casual drinker	2.167 (1.118–4.199)	0.022	0.963 (0.390–2.375)	0.934
Problematic drinker	0.157 (0.021–1.205)	0.075	0.117 (0.014–0.943)	0.044
Women, ≥65 years (*n* = 43)				
Abstainer	1.000		1.000	
Casual drinker	0.210 (0.101–0.349)	&lt; 0.001	0.349 (0.153–0.792)	0.012
Problematic drinker	0.999 (0.000–9.999)	0.999	0.999 (0.000–9.999)	0.999

*DM* diabetes mellitus, *OR* odds ratio

Approximately 56% of the total participants were subjected to abPWV and cIMT measurements. A demographic comparison between the groups in whom abPWV was measured (+) vs. not measured (−) and cIMT was measured (+) vs. not measured (−) are presented in the Additional file 1: Table S1. The abPWV (+) group consisted of a slightly lower number of men (65%) than that in the abPWV (−) group (77%), and the age in the cIMP (+) group was slightly lower (51 ± 11 years) than that in the cIMP (−) group (54 ± 10 years). Other factors such as DM, hypertension, and smoking were not different between the groups. The linear regression model analysis was performed to assess the correlation between drinking patterns and abPWV and cIMT (Table 3). Casual drinking was significantly associated with a lower abPWV and thinner carotid intima-media in both with and without adjustment of DM/hypertension; however, the predictive power was lost when age and sex were adjusted for in addition to DM and hypertension.

**Table 3:** Linear regression model for the ankle-brachial pulse wave velocity and carotid intima-media thickness to assess the independent effects of alcohol consumption

	Unadjusted		Adjusted for DM and hypertension		Adjusted for DM, hypertension, age, and sex	
	beta	*p*-value	beta	*p*-value	beta	*p*-value
Ankle-brachial pulse wave velocity (*n* = 569)						
Casual drinker	−0.183	&lt; 0.001	−0.154	0.002	−0.964	0.335
Problematic drinker	− 0.001	&lt; 0.001	− 0.471	0.638	0.928	0.354
Carotid intima-media thickness (*n* = 562)						
Casual drinker	−0.131	0.013	−0.118	0.025	0.001	0.981
Problematic drinker	−0.037	0.422	−0.45	0.330	−0.008	0.860

## Discussion

### Salient findings

In the current cross-sectional study, we found that casual drinkers have less severe CV organ damages in the subclinical stages than abstainers or problematic drinkers, which support the “V- or J-shaped” association between alcohol consumption and atherosclerosis. The favorable effects of casual drinking on CAC deposits were remarkable even in subjects with known risk factors for CV diseases, such as DM, hypertension, smoking, and fatty liver. The results of the current investigation are novel because: 1) it was performed in a Korean population whose drinking pattern and taste for liquors are different from those of Westerners; and 2) subclinical CV organ damages were comprehensively assessed using recent diagnostic tools in subjects with no history of CV diseases. Our results would serve as an additional evidence for the French paradox, an old epidemiologic observation.

### Favorable effects of alcohol on CV diseases: truth or fallacy?

There is an accumulating evidence of a J-shaped association between the amount of alcohol consumption and prevalence/mortality of CV diseases [3–5, 12, 13]. In the current investigation, we also observed a similar phenomenon, i.e., a V-shaped association in the multiple subgroups with risk factors of CAC deposits (Fig. 1). Drinking habits are influenced by age and sex for biological, behavioral, and/or social reasons. Therefore, we assessed the correlation between alcohol drinking and CAC deposits separately in groups of different age and sex (Table 2). Problematic drinking seemed to exert hazardous effects on the CV system in men, but was rather favorable in women aged &lt; 65 years. Conversely, casual drinking appeared to be noticeably favorable to CV conditions in both men and women aged ≥65 years. These suggest that the following would play a role as confounding factors: 1) problematic drinking was relatively uncommon in women, and they comprised a small portion of our cohort; and/or 2) the definition for problematic drinking was too strict in women (&gt; 7 standard drinking/week). Nishiwaki et al. reported that acute alcohol ingestion reduced arterial stiffness in the normal population [14]; our data exhibited a chronically lower abPWV in casual drinkers even in the DM/hypertension-adjusted analysis than those in the abstainers. However, the independent predictive power of casual drinking became insignificant when age and sex were adjusted in the linear regression model for abPWV in addition to DM and hypertension. The statistical results of the cIMT were also similar (Table 3). These findings imply that alcohol drinking is only a modest factor of vascular softening and atherosclerosis prevention compared with other strong factors, such as age and comorbidities.

The proportion of fatty liver (presumably non-alcoholic fatty liver disease [NAFLD]) was lower in casual drinkers than in abstainers and problematic drinkers. However, a J-shaped association was exhibited between the parameters associated with fatty liver and amount of alcohol consumption. The effects of light/moderate alcohol consumption on NAFLD is also an issue among studies that reported a positive [15–17] or negative association [18], as well as a J-shaped association, with the amount of alcohol consumption [19]. Our study may support the J-shaped association. The ultrasound used in our study might not be a sensitive method to assess the exact condition of the liver.

The potential benefit of casual drinking is still inconclusive [5]; some authors even reported or claimed no association [20–23]. Conversely, the binge drinking-related problem is clearly one of the biggest health/social issues in the modern world [5, 24]. Thus, we are also skeptical on using alcohol as a CV medicine just based on several cross-sectional data, including ours. Nevertheless, we observed that casual drinkers seem to be less vulnerable to subclinical organ damage at all events; thus, universal discouragement of potentially healthful drinking should be scrutinized.

### Why is drinking potentially cardioprotective, owing to alcohol itself or other healthful substances in beverages?

The mechanisms for the potential cardioprotective effects of drinking have not been fully elucidated to date. Some authors have claimed that a low prevalence of CV diseases observed in wine or beer drinkers might be associated with the antioxidants contained in the beverages [5, 7, 8]. Meanwhile, others have suggested the beneficial effects of alcohol itself [25]. To clarify this question, a well-designed study in a large population is required. However, there is a fundamental restriction in revealing the long-term cardioprotective effects of alcohol because a longitudinal randomized controlled study on pure alcohol is technically impossible [5, 20, 26]. Thus, more evidence from various ethnic groups and countries with different drinking cultures would be informative. One strong point of our study is that the entire cohort consisted of Koreans who were underrepresented in previous epidemiologic investigations and whose drinking pattern is distinct from those of the Westerners; the potential cardioprotection of alcohol has been largely claimed based on the results of studies from Western countries [2, 4, 12, 13, 20, 24, 26, 27]. Binge drinking is more common than everyday-small-amount drinking in Korea, and the most frequently consumed alcoholic beverage is a type of distilled alcohol that contains almost zero antioxidants. Therefore, the favorable effects of drinking presented in the current study may be from alcohol itself.

### Theoretical hypothesis for the current results and required further animal studies

Based on this study, there are several hypotheses for the potential mechanism for the cardioprotective effect of alcohol. Drinking raises the HDL level [28], which was suggested to play an anti-atherosclerotic role. Alcohol inhibits platelet aggregation [29]. Light-to-moderate drinking is known to have anti-inflammatory effects [27, 28] and to improve insulin resistance [5, 30]. Even the genetic predisposition of less alcohol consumption was suggested as a confounding factor for the fallacy of the cardioprotection of alcohol [20]. However, these hypotheses are not widely validated. Lipid profiles, insulin sensitivity, inflammation, hormonal status, and hemodynamic changes can affect CV conditions, which can be affected by alcohol. Providing various amounts of alcohol on animal models of atherosclerosis would help answer the questions in parallel with human studies to confirm these effects. Atherosclerosis is a chronic inflammatory disease, for which endothelial cells and inflammatory cells as well as lipid profiles and LDL oxidation exert. To determine the role of alcohol, its effects on cellular and molecular changes should be studied. Eicosanoids are also crucial factors in inflammation and clotting; thus, the roles of alcohol on their release from inflammatory cells, endothelial cells, and platelets need to be determined.

### Limitations

First, the samples in this study were people presenting to a hospital for health check-up. This limits the generalizability of the current investigation. Second, this study has an inevitable limitation of cross-sectional analyses. Therefore, the causal relationship of alcohol with CV organ damages cannot be determined. Third, the beverage type (spirit, wine, beer, etc.), drinking pattern (regular or irregular), and drinking duration were not assessed in detail. Alcohol consumption was semi-quantitatively analyzed. Fourth, the treatment and medications for comorbidities, such as DM and hypertension, were not included in the data collection. Finally, the abPWV and cIMT were assessed in only approximately half of the cohort.

## Conclusions

We performed cross-sectional analyses to investigate the effects of habitual alcohol drinking on the subclinical CV morbidity in healthy Korean subjects with no apparent CV diseases. Compared with abstinence and problematic drinking, casual drinking was associated with less severe CV organ damages in the subclinical stages.

## Additional file

Additional file 1: **Table S1.** Demographic Comparison between the Groups Subjected to abPWV and cIMT Measurement. Description of data: Demographic comparison between the groups in whom abPWV was measured (+) vs. not measured (−) and cIMP was measured (+) vs. not measured (−) are presented. (DOCX 13 kb)

## Abbreviations

abPWV: Ankle-brachial pulse wave velocity
BP: Blood pressure
CAC: Coronary artery calcium
CI: Confidence interval
cIMT: Carotid intima-media thickness
CT: Computed tomography
CV: Cardiovascular
DM: Diabetes mellitus
ECG: Electrocardiogram
HDL: High-density lipoprotein
LDL: Low-density lipoprotein
NAFLD: Non-alcoholic fatty liver disease
OR: Odds ratio
PWV: Pulse wave velocity
SD: Standard deviation
