# Supplementary material for: Casual alcohol consumption is associated with less subclinical cardiovascular organ damage in Koreans: a cross-sectional study
Source: BMC Public Health. 2018 Sep 4;18:1091. doi: 10.1186/s12889-018-6000-x (PMC6123993; doi:10.1186/s12889-018-6000-x)
Supplement: Supplementary file 1 — Table S1. Demographic Comparison between the Groups Subjected to abPWV and cIMT Measurement. Description of data: Demographic comparison between the groups in whom abPWV was measured (+) vs. not measured (−) and cIMP was measured (+) vs. not measured (−) are presented. (DOCX 13 kb) [file 12889_2018_6000_MOESM1_ESM.docx]

**Additional file 1: Table S1.** Demographic Comparison between the Groups Subjected to abPWV and cIMT Measurement

| Variables |  |  | *p*-value |
| --- | --- | --- | --- |
| ***abPWV*** | (+)  (n=569) | (-)  (n=435) |  |
| Age, years | 53±10 | 53±11 | *p*=0.873 |
| Male sex, n (%) | 389 (65) | 332 (77) | *p* =0.006 |
| Diabetes mellitus, n (%) | 44 (8) | 48 (11) | *p*=0.077 |
| Hypertension, n (%) | 137 (24) | 117 (39) | *p*=0.306 |
| Smoking, n (%) | 262 (46) | 225 (52) | *p*=0.074 |
| ***cIMT*** | (+)  (n=562) | (-)  (n=442) |  |
| Age, years | 51±11 | 54±10 | *p*=0.001 |
| Male sex, n (%) | 395 (70) | 326 (74) | *p* =0.231 |
| Diabetes mellitus, n (%) | 55 (10) | 48 (8) | *p*=0.509 |
| Hypertension, n (%) | 146 (26) | 108 (25) | *p*=0.609 |
| Smoking, n (%) | 280 (50) | 207 (47) | *p*=0.372 |
